# Structural and Spectroscopic Analysis of the Kinase Inhibitor Bosutinib and an Isomer of Bosutinib Binding to the Abl Tyrosine Kinase Domain

**DOI:** 10.1371/journal.pone.0029828

**Published:** 2012-04-06

**Authors:** Nicholas M. Levinson, Steven G. Boxer

**Affiliations:** Department of Chemistry, Stanford University, Stanford, California, United States of America; Medical College of Wisconsin, United States of America

## Abstract

Chronic myeloid leukemia (CML) is caused by the kinase activity of the BCR-Abl fusion protein. The Abl inhibitors imatinib, nilotinib and dasatinib are currently used to treat CML, but resistance to these inhibitors is a significant clinical problem. The kinase inhibitor bosutinib has shown efficacy in clinical trials for imatinib-resistant CML, but its binding mode is unknown. We present the 2.4 Å structure of bosutinib bound to the kinase domain of Abl, which explains the inhibitor's activity against several imatinib-resistant mutants, and reveals that similar inhibitors that lack a nitrile moiety could be effective against the common T315I mutant. We also report that two distinct chemical compounds are currently being sold under the name “bosutinib”, and report spectroscopic and structural characterizations of both. We show that the fluorescence properties of these compounds allow inhibitor binding to be measured quantitatively, and that the infrared absorption of the nitrile group reveals a different electrostatic environment in the conserved ATP-binding sites of Abl and Src kinases. Exploiting such differences could lead to inhibitors with improved selectivity.

## Introduction

Chronic myeloid leukemia (CML) is the result of the constitutive kinase activity of the tyrosine kinase BCR-Abl, the product of the *bcr-abl* gene fusion present on the Philadelphia chromosomes of patients with CML [1]. Imatinib is a selective inhibitor of BCR-Abl, and the introduction of imatinib into the clinic represented a dramatic improvement in CML therapy [2]. The tyrosine kinases c-Kit and platelet derived growth factor receptor (PDGFR) are also potently inhibited by imatinib, which is now used to treat malignancies caused by dysregulated forms of these proteins [3], [4].

Despite the success of imatinib in treating CML, some patients ultimately develop resistance to imatinib treatment and undergo clinical relapse [5]. Although *bcr-abl* gene amplification has been observed, resistance is most often caused by point mutations in the kinase domain of BCR-Abl that abrogate the binding of imatinib [5], . The emergence of imatinib resistance has led to a search for additional inhibitors of BCR-Abl, and the second generation inhibitors dasatinib and nilotinib were recently approved for use in CML patients resistant to imatinib, as well as for front-line therapy [8], [9].

While dasatinib and nilotinib are active against most imatinib-resistant BCR-Abl mutations, neither drug is effective against BCR-Abl bearing the common T315I mutation. Patients that initially respond to dasatinib therapy and subsequently relapse have been shown to possess new BCR-Abl mutations, indicating that clinical resistance to second-generation inhibitors can emerge [10]. There is therefore continued interest in obtaining additional Abl inhibitors, both to combat resistance and to broaden the therapeutic options for CML patients.

Bosutinib is a second-generation dual Abl/Src inhibitor that exhibits potent growth inhibition of CML cells *in vitro*, is active against multiple imatinib-resistant BCR-Abl mutations and has demonstrated efficacy in ongoing clinical trials for imatinib-resistant CML [11], [12], [13]. Bosutinib is devoid of activity against the receptor tyrosine kinases Kit and PDGFR, and, like other next generation BCR-Abl inhibitors, is a more potent inhibitor of Abl than imatinib [11], [14]. Due to its activity against the Src kinases, bosutinib has shown efficacy against several types of cancer in which Src is implicated [15], [16]. Bosutinib is a 4-anilinoquinoline-3-carbonitrile inhibitor (see Fig. S2A for structure) that is similar in structure to the drugs erlotinib and gefitinib, inhibitors of the epidermal growth factor receptor (EGFR). Crystal structures of other inhibitors of this class bound to kinases have been solved, but the details of the interaction between bosutinib and Abl are unknown. In the course of studies of electrostatic interactions in the ATP-binding sites of several kinases, briefly outlined at the end of this report, we determined the crystal structure of the kinase domain of Abl bound to bosutinib at 2.4 angstrom resolution. The structure explains the effects of imatinib resistance mutations on bosutinib binding, and provides a basis for interpreting spectroscopic measurements that probe the environment of the ATP-binding site of Abl and other kinases.

## Materials and Methods

### Protein purification and crystallization

The kinase domains of wild-type human c-Abl (residues 229–512) and wild-type and T338I human c-Src (residues 254–536) were expressed in *E.coli* BL21 (DE3) (Invitrogen) and purified by affinity, ion exchange and gel filtration chromatography as previously described [17]. Extensive previous work has demonstrated that Abl expressed in bacteria is correctly folded and retains catalytic activity [17], [18], [19], [20]. Samples of the Abl:bosutinib and Abl:bosutinib isomer complexes were prepared by mixing Abl kinase domain (in sample buffer: 50 mM Tris-HCl pH 8.0, 150 mM NaCl, 2 mM DTT) with a three-fold excess of bosutinib (Tocris Bioscience) or bosutinib isomer (LC Labs) in DMSO and performing buffer exchange with sample buffer to remove the DMSO and unbound drug. Sparse matrix screening was used to identify conditions conducive to crystallization. Crystals were obtained in 0.1 M Ammonium Acetate, 0.1 M MES pH 5.5 and 11% PEG 10 K, and cryo-protected in the same condition plus 30% glycerol.

### Kinase assays

Kinase activity was measured using a coupled kinase assay in which the production of ADP is linked to the oxidation of NADH by pyruvate kinase and lactate dehydrogenase [17]. Assays were performed in 75 µl reactions containing 100 mM Tris-HCl pH 8.0, 10 mM MgCl_2_, 2 mM ATP (Sigma Aldrich), 0.5 mM Abltide substrate peptide (Anaspec), 1 mM phosphoenolpyruvate (Sigma Aldrich), 0.6 mg/ml NADH (Sigma Aldrich), 1 mM DTT, and 50 nM Abl kinase. The measurements were corrected for background activity in the absence of substrate peptide.

### X-ray data collection and refinement

X-ray diffraction data were collected at the Stanford Linear Accelerator Center on beamlines 12-2 and 7-1. Data were processed with mosflm [21] and CCP4 [22]. The structure of Abl bound to the bosutinib isomer was solved by molecular replacement in Phenix [23] using the structure of Abl bound to VX-680 [24](pdb code 2F4J) as a search model. Model rebuilding was performed with Coot [25] and refinement with Phenix. For the structure of Abl bound to authentic bosutinib, the R-free flags used in refinement were copied from the bosutinib isomer dataset, and the refined model of Abl bound to the bosutinib isomer was used with only limited refinement. To confirm the positions of the chlorine atoms on the aniline ring of bosutinib we exploited the anomalous scattering of chlorine. While the chlorine K absorption edge is near that of sulfur at ∼2800 eV (4.4 Å), chlorine and sulfur both retain significant anomalous scattering at shorter wavelengths [26]. Using synchrotron radiation at a wavelength of 1.76 angstroms, the longest wavelength accessible on beamline 7-1 at the Stanford Synchrotron Radiation Laboratory, we collected a highly redundant dataset to a resolution of 2.9 angstroms on a crystal of the Abl:bosutinib complex (Table 1). Anomalous difference maps calculated from this data using the phases from the refined 2.4 angstrom structure showed strong peaks (∼5 standard deviations above the mean) for many of the sulfur atoms in the protein, as well as the four chlorine atoms of the two bosutinib molecules in the asymmetric unit.

**Table 1:** Data collection and refinement statistics.

Data Collection	Abl:bosutinib isomer	Abl:bosutinib	Abl:bosutinib (anomalous)
X-ray wavelength (Å)	0.98	0.98	1.76
Space group	P22_1_2_1_	P22_1_2_1_	P22_1_2_1_
Unit cell dimensions (Å)	57.3,113.6,128.4	56.9,113.8,127.6	57.3,113.6,128.4
Resolution range (Å)	28-2.9	63-2.4	63-2.9
R_sym_ a	0.144 (0.657)	0.112 (0.552)	0.128 (0.579)
Average I/σ(I)^a^	8.8 (2.6)	13.5 (3.1)	15.7 (5.2)
Completeness^a^	97.4% (98.9%)	93.8 (94.8)	99.3 (98.5)
Redundency	4.1	5.9	13.7
**Refinement**			
Number of reflections	18388	30158	
R_work_/R_free_	0.178/0.260	0.188/0.249	
# of protein atoms	4350	4306	
# of ligand atoms	130	130	
# of solvent atoms	79	152	
RMSD Bond lengths (Å)	0.008	0.008	
Bond angles (°)	1.112	1.059	

a values in parentheses are for the highest resolution shell.

### Fluorescence binding assays

Abl and Src kinase domain (5 nM) were mixed with different concentrations of bosutinib in 20 mM Tris-HCl pH 8.0, and the fluorescence emission was monitored at 480 nm, with excitation at either 280 nm or 350 nm. For the T338I mutant of Src the fluorescence emission intensity was plotted as a function of the bosutinib concentration and fit to a single binding site model (Graphpad Prism) to obtain the equilibrium dissociation constant. For wildtype Abl and Src the binding is too tight to determine in this manner. Instead, the titrations were fit directly to the analytical solution to the one-to-one binding equilibrium using Mathematica (Wolfram Research).

Like bosutinib, vandetanib exhibits a strong increase in fluorescence on binding to Src and Abl. Binding curves, where the emission intensity at 440 nm (with excitation at 280 nm) was plotted as a function of the total vandetinib concentration, were fit to a single binding site model with Graphpad Prism.

To measure bosutinib binding to phosphorylated Abl, Abl kinase domain (100 µM) was phosphorylated with Hck kinase domain (5 µM), in 2 mM ATP, 10 mM MgCl_2_, 20 mM Tris-HCl pH 8.0 and 10% glycerol, for 5 hours at room temperature, and phosphorylation was verified by mass spectrometry. Experiments were performed in parallel with the phosphorylated and unphosphorylated samples.

### Infrared spectroscopy

Samples of the kinase:inhibitor complexes were prepared by mixing kinase in buffer (50 mM Tris-HCl pH 8.0, 150 mM NaCl, 2 mM DTT, 10% glycerol) and inhibitor stocks in DMSO to a final DMSO concentration of 5% and concentrating the samples to ∼2 mM. Due to the very tight binding as well as the very low aqueous solubility of the inhibitors, the concentration of free ligand in these samples was negligible. Samples were loaded into a sample cell with ∼100 µm path length and infrared spectra were measured using a Vertex FTIR spectrometer (Bruker).

The linear Stark tuning rate of the nitrile goup of bosutinib and the bosutinib isomer were determined as described previously [27]. Briefly, the compounds were dissolved in 1-propanol at 50 mM concentration and loaded into a custom sample cell consisting of nickel-coated sapphire windows. Samples were flash-frozen in a custom-built liquid nitrogen immersion cryostat [28], and a high voltage power supply was used to apply an external electric field across the sample. Stark spectra are the difference in the absorbance spectra with the applied field on and off, which were each determined from the average of 128 scans of the interferometer mirror. The linear Stark tuning rate was determined from a numerical fit of the derivatives of the absorbance to the Stark spectrum.

### NMR spectroscopy

Samples of bosutinib (Tocris Bioscience) and the bosutinib isomer (LC Labs) were dissolved in DMSO-*d_6_* to a concentration of 20 mM. 1-dimensional proton and carbon spectra and 2-dimensional ^1^H-^13^C Heteronuclear Single Quantum Coherence (HSQC) experiments were recorded on 500 and 600 MHz NMR spectrometers.

### Accession numbers

Structure factors and the coordinates of the Abl:bosutinib structure have been deposited in the Protein Data Bank (http://www.rcsb.org) with accession number 3UE4.

## Results and Discussion

### Identification of a reliable commercial source of bosutinib

**Figure 1:**
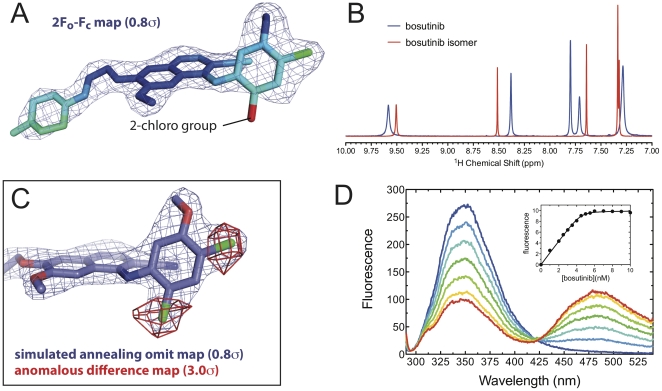
Identification of two different isomers of bosutinib. A) View of the ligand from our initial structure of Abl bound to the bosutinib isomer. The ligand is shown as sticks, colored according to the temperature factors (B-factors) of the atoms, with blue indicating low B-factors and red indicating high B-factors. The 2F_o_-F_c_ electron density map, calculated with phases derived from a refined molecular model that included the 2-chloro group of bosutinib, is shown as a blue mesh. B) ^1^H NMR spectra of bosutinib (Tocris Bioscience, blue) and the bosutinib isomer (LC Labs, red), showing only the aromatic region. C) View of the ligand from our structure of Abl bound to authentic bosutinib. The coordinates of bosutinib are shown as blue sticks. A simulated annealing omit map contoured at 0.8 standard deviations above the mean (0.8σ) is shown as a blue mesh. An anomalous difference map, contoured at 3.0σ, is shown in red. D) Fluorescence emission spectra (excitation at 280 nm) of 50 nM Abl kinase domain in the presence of varying concentrations of bosutinib (the spectra are colored according to bosutinib concentration, which was varied in 10 nM increments from 0 nM shown in blue to 60 nM shown in red). The inset shows a binding curve measured for 5 nM Abl. The normalized fluorescence intensity at 480 nm is plotted as a function of the total bosutinib concentration. The smooth line shows the numerical fit (see Materials and Methods).

The kinase domain of human Abl was expressed in bacteria, and purified to homogeneity. Kinase assays demonstrated that, as previously reported, the bacterially expressed protein is catalytically active (Figure S1) [17]. We co-crystallized Abl with a sample of “bosutinib” obtained from the company LC Labs (also known as PKC Pharmaceuticals), and solved the structure by molecular replacement to 2.9 angstroms resolution (Table 1). During the course of the refinement of this structure we noticed a peculiar lack of electron density for the 2-chloro atom on the aniline ring of the small molecule, which called into question the identity of the compound (Figure 1A). A series of NMR experiments demonstrated that two different isomers, that differ in the position of substituents on the aniline ring, are being sold under the name “bosutinib” by different vendors, and that the compound initially used was, in fact, an isomer of bosutinib. These experiments are discussed in detail in the Supporting Information (Figure S2), and a brief description is given below.

Mass spectrometry showed that the compounds from both commercial sources had the expected mass of *m/z* 530.1 (M+1). While the proton NMR spectra of the compound sold by Tocris Bioscience precisely matches that reported for bosutinib by the research group at Wyeth that developed the drug [29], the spectrum of the compound sold by LC Labs is significantly different in the aromatic region (Figure 1B). Multidimensional NMR experiments on the LC Labs compound (Figure S2) indicated the presence of C2 symmetry on the aniline ring, which is incompatible with the chemical structure of bosutinib, and suggested that the positions of the substituents on the aniline ring (two chlorine atoms and a methoxy group) were what differed between the two compounds.

Crystals of Abl kinase domain bound to the compound from Tocris Bioscience were obtained in the same crystal form, and we solved the structure to a resolution of 2.4 angstroms (Table 1). A simulated annealing omit map shows excellent electron density for the drug, with the aniline ring clearly resolved (Figure 1C). However, because the chlorine atoms and methoxy group on the aniline ring each possess 17 electrons, the x-ray scattering from these groups is similar, and at the resolution of this structure they cannot be distinguished from each other. To conclusively demonstrate that these substituents were correctly positioned on the aniline ring, we exploited the anomalous scattering of chlorine. X-ray diffraction data were collected using a synchrotron x-ray wavelength of 1.76 angstroms, where the anomalous scattering of chlorine is significant (Table 1) [26]. Anomalous difference maps calculated using the phases from the refined molecular model show strong peaks (greater than 4 standard deviations above the mean) for the chlorine atoms of the drug in the ortho and para positions on the aniline ring, confirming the identity of the compound (Figure 1C).

We refer to the correct compound as “bosutinib” and to the incorrect one as the “bosutinib isomer”. To date the protein databank contains two entries for “bosutinib”, bound to calcium calmodulin regulated protein kinase II and to serine threonine kinase 10. The title of the latter entry states that the compound was modified by radiation damage, and the pdb coordinates (pdb code 3ZZ2) show that the 2-chloro atom on the aniline ring is missing, and a chlorine atom is instead located in the meta position. Given the fact that the affected atom is the same one that is missing in our bosutinib isomer, a likely possibility is that the authors were afflicted by the same problem we have encountered. Interestingly, the ^1^H NMR spectrum of our bosutinib isomer is very similar to the NMR spectrum reported in a paper describing an alternative synthesis for bosutinib [30]. These observations raise the prospect that this problem is widespread, with multiple vendors selling the incorrect isomer of bosutinib.

The structures of Abl bound to the two different compounds are almost identical, although the bosutinib complex exhibits considerably lower temperature factors, and the amino acid side chains in contact with the small molecule are better resolved in the bosutinib complex. All spectroscopic measurements reported in this work were performed on both compounds. Below we focus on the data obtained with authentic bosutinib; further results with the incorrect isomer can be found in the supporting information.

### Bosutinib becomes strongly fluorescent upon binding to Abl or Src kinases

In the course of studying the interaction between bosutinib and the kinases Src and Abl, we discovered that the inhibitor becomes strongly fluorescent upon binding to these proteins, a property that could be of general utility for measuring inhibitor binding. Both bosutinib and the bosutinib isomer possess an absorption band at 350 nm, and excitation of the free ligand at 350 nm results in weak fluorescence emission at 480 nm. Upon binding to Src or Abl, the fluorescence intensity at 480 nm increases ∼10 fold. In the case of the bosutinib isomer the background fluorescence of the free compound is even lower, and the relative increase in fluorescence on binding is ∼500 fold. Interestingly, for both compounds, excitation of the protein:inhibitor sample at 280 nm also results in strong fluorescence emission at 480 nm. A titration of bosutinib with excitation at 280 nm results in quenching of tryptophan fluorescence at 340 nm and a rise in emission at 480 nm, indicating that Förster resonance energy transfer (FRET) occurs between the protein and bosutinib (Figure 1D).

The increase in inhibitor fluorescence upon binding affords a convenient assay for quantifying inhibitor binding, which we used to measure the binding constants of bosutinib for Src and Abl kinases. Because the minimum concentration of fluorescent protein:ligand complex that can be reliably measured on our fluorimeter is ∼1 nM, and the binding is considerably tighter than this, the titration curves were fitted using a numerical fitting procedure that accounts for ligand depletion (Figure 1D inset, see Materials and Methods for the fitting procedure). The binding constants for Src and Abl are both ∼200 picomolar.

### Interactions between bosutinib and Abl

**Figure 2:**
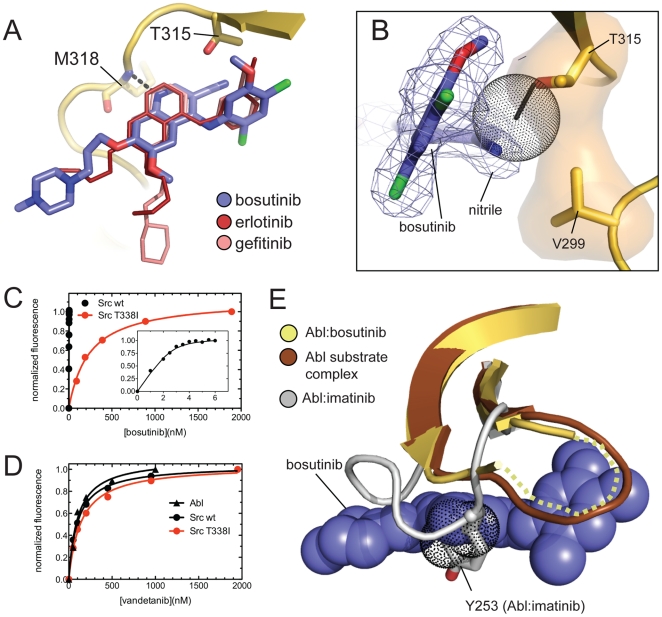
Structure of authentic bosutinib bound to the Abl tyrosine kinase domain. A) Interaction of bosutinib (blue) with the hinge region of Abl (yellow). For comparison, the binding modes of erlotinib (red) and gefitinib (pink) are also shown, and were obtained by aligning the structures of these compounds bound to EGFR (pdb codes 1M17 and 2ITY for erlotinib and gefitinib, respectively) on the hinge region of Abl. B) The interactions between bosutinib and T315 and V299 of Abl are shown. The residues T315 and V299 are shown as sticks and a yellow surface, and bosutinib is shown as blue sticks, with the 2F_o_-F_c_ electron density map shown as a blue mesh. The T315I mutation is modeled as thin black sticks, and the resulting clash with bosutinib is shown as black dots. C) Binding curves for bosutinib binding to Src and the Src T338I mutant. The fluorescence intensity measured at 480 nm is plotted as a function of the total bosutinib concentration. The inset shows an expanded view of the binding curve for Src. The equilibrium dissociation constants were determined by a fitting procedure described in the Materials and Methods. D) Binding curves for vandetanib binding to Abl, Src and the Src T338I mutant. The fluorescence emission intensity measured at 440 nm, with excitation at 280 nm, is plotted as a function of the total vandetanib concentration. E) The conformation of the P-loop in our structure (shown in yellow, the two disordered residues are indicated as a dashed yellow line), compared to that observed in the imatinib cocrystal structure (pdb code 1IEP, shown in gray), and a substrate complex of Abl (pdb code 2G1T, shown in brown). The clash between Y253 and bosutinib that would result from the collapsed conformation of the P-loop is shown as black dots.

The asymmetric unit of our structure contains two copies of the Abl: bosutinib complex (labeled A and B in the molecular model), which are almost identical, and only complex B will be discussed. Bosutinib occupies the ATP-binding site of Abl, sandwiched between the N-terminal and C-terminal lobes of the kinase. The binding mode is very similar to that observed with the chemically related inhibitors gefitinib and erlotinib bound to EGFR [31], [32], with the quinoline group of bosutinib oriented in almost identical fashion to the quinazoline groups of the EGFR inhibitors, except for a slight rotation of the quinoline plane to accommodate the nitrile group, which would otherwise clash with the sidechain of T315 (Figure 2A). The only hydrogen bond formed between bosutinib and Abl is between the quinoline N1 nitrogen atom and the backbone amide of M318 (a residue in the hinge region of the kinase), a characteristic feature of the binding mode of this class of inhibitors. The 2,4-dichloro-5-methoxy aniline fragment of bosutinib is oriented at a ∼65° angle to the plane of the quinoline heterocycle and fills a hydrophobic pocket formed by residues projecting from the N-lobe into the ATP-binding site. The flexible N-propoxy-N-methylpiperazine group is well ordered in our structure and extends out of the ATP-binding site, where it makes van der Waals contacts with the kinase hinge region.

Of the three BCR-Abl inhibitors currently approved for clinical use, the interaction of bosutinib with Abl is most similar to that of dasatinib [33], but in the deepest portion of the ATP-binding site there are notable differences. In the dasatinib cocrystal structure an amide group on dasatinib forms a hydrogen bond to the sidechain hydroxyl of T315. The nitrile group of bosutinib occupies the same space as the amide of dasatinib, but is at an angle incompatible with a hydrogen bond and makes only van der Waals contacts with T315. The aniline substituent of bosutinib is bound in a similar orientation as the 2-chloro-6-methyl phenyl group of dasatinib, but displaced ∼2 angstroms further out of the ATP-binding site towards the phosphate-binding loop. There is thus a cavity where the 2-chloro-6-methyl phenyl ring of dasatinib would reside, which is filled by an ordered water molecule in the bosutinib complex.

### Implications for the activity of bosutinib against imatinib-resistant BCR-Abl mutants

The interactions between bosutinib and the ATP-binding site of Abl explain the effect of several imatinib-resistance mutations on bosutinib binding [34]. The sidechain of T315 is completely enveloped by bosutinib, making extensive van der Waals contacts with both the nitrile group and the 5-methoxy group of the aniline ring (Figure 2B). The nitrile group is also in van der Waals contact with the sidechain of V299. Both the T315I and V299L mutations would result in steric clashes with bosutinib, explaining why these mutations confer resistance to bosutinib [34]. We used our fluorescence binding assay to measure the binding constant for the gatekeeper mutant of Src (T338I), which, unlike the T315I mutant of Abl, expresses well in bacteria, and found that the binding is much weaker than for wildtype Src, with a K_D_ value of ∼250 nM (Figure 2C).

Despite the extent of the contacts between T315 and bosutinib, modeling indicates that the only clash that results between bosutinib and the isoleucine residue of the T315I mutant is with the nitrile group, suggesting that the drug could be accommodated by this mutant if the nitrile group were missing (Figure 2B). Thus the binding of 4-anilinoquinazolines, which are similar to bosutinib but lack the nitrile group, should not be impeded by the T315I mutation. Indeed, in a screen of inhibitors against a large panel of kinases the 4-anilinoquinazolines erlotinib and CI-1033 inhibited wildtype Abl and the T315I mutant with similar K_D_ values [35]. To further test this hypothesis, we used our fluorescence binding assay to measure the binding of the 4-anilinoquinazoline vandetanib, a drug that is used in the treatment of medullary thyroid cancer, caused by dysregulated RET tyrosine kinase [36]. Indeed, we found that vandetanib inhibits Abl, Src, and the Src T338I mutant with very similar K_D_ values of ∼100 nM (Figure 2D).

While these 4-anilinoquinazolines inhibit Abl too poorly to be effective in cells, where they must compete with high concentrations of ATP for binding to the kinase, other 4-anilinoquinazolines could prove effective against the T315I mutation. It is interesting to note that, during the treatment of cancers caused by dysregulated EGFR with 4-anilinoquinazoline inhibitors, clinical resistance is caused by mutation of the gatekeeper threonine residue to methionine [37], but that this mutation exerts its effect not through steric hindrance, but through lowering the K_M_ value for ATP [38]. It appears that the inclusion of the nitrile group in bosutinib, which improves the potency against wildtype Src kinase relative to the corresponding quinazoline [39], inadvertently made the inhibitor highly susceptible to resistance mediated by mutation of the gatekeeper residue.

Our structure also explains the ability of bosutinib to override imatinib resistance mutations that map to the phosphate-binding loop (P-loop), a loop involved in binding the phosphates of ATP. It has been argued that these P-loop mutations exert their effects by destabilizing the conformation of the P-loop favored by imatinib, in which the loop collapses to form a hydrophobic cage that envelops the drug [6], [40], [41]. Structures of Abl bound to other kinase inhibitors have shown similar collapsed P-loop conformations [24], [42], suggesting that the P-loop of Abl is particularly susceptible to conformational changes induced by the binding of inhibitors. In our structure of Abl bound to bosutinib two residues at the tip of the P-loop (Q252 and Y253) are poorly ordered, but the remainder of the loop adopts an extended conformation similar to the β-hairpin observed in a substrate complex of Abl [43] and makes no contacts with bosutinib (Figure 2E). Aligning the structure of Abl in complex with imatinib onto our structure reveals that the collapsed conformation of the P-loop is incompatible with bosutinib binding, as it would produce a clash between the sidechain of Y253 and the 6-methoxy group of bosutinib (Figure 2E). In an *in vitro* study of the effect of imatinib resistance mutations on inhibition by dasatinib, nilotinib and bosutinib, bosutinib was not affected by either the Q252H or Y253F mutations [34], consistent with the lack of interactions between bosutinib and the P-loop.

### The DFG motif adopts an inactive conformation in our structure

**Figure 3:**
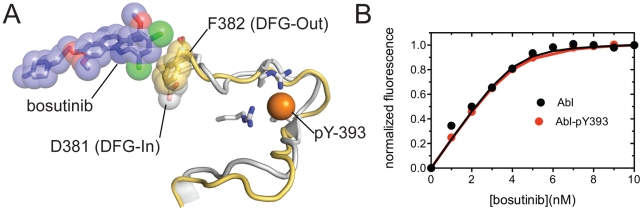
Bosutinib binds to both DFG-In and DFG-Out Abl. A) Comparison of the conformation of the activation loop and DFG motif in our structure (DFG-Out, yellow) and in the dasatinib cocrystal structure (DFG-In, gray). The sidechains of D381 in the dasatinib structure and F382 in our structure, which occupy very similar positions, are shown as spheres. Bosutinib is shown as sticks and spheres. The position of the phosphate group on the phosphorylated sidechain of Y393 in the dasatinib structure is shown as an orange sphere. B) Binding curves for bosutinib binding to Abl and to Abl phosphorylated on the activation loop (Abl-pY393).

The kinase inhibitor imatinib binds to an inactive conformation of Abl in which the aspartate and phenylalanine residues of the catalytically important Aspartate-Phenylalanine-Glycine (DFG) motif exchange positions (called the DFG-Out conformation, in contrast to the active DFG-In conformation) [40], [42]. In contrast, several crystal structures, including structures of gefitinib and erlotinib bound to EGFR, have demonstrated that 4-anilinoquinazoline inhibitors usually bind to the active conformations of protein kinases [31], [32]. In our structure of Abl bound to bosutinib, the DFG motif is in an inactive DFG-Out conformation, but this conformation is distinct from the DFG-Out conformation observed in complex with imatinib. In the structure of Abl bound to imatinib, the activation loop undergoes a dramatic rearrangement from the active conformation in which the C-terminal portion of the loop blocks the active site, resulting in a ∼4 Å shift of the DFG motif nearer to the front of the active site. In our structure the overall conformation of the activation loop is instead similar to that observed in active kinases, except for the conformation of the DFG motif itself, as well as a single-residue shift in the register of the short β–sheet in the N-terminal portion of the loop (residues 383–386). This conformation of the activation loop has been observed previously in structures of Abl bound to the kinase inhibitors PD16 and PD17 [42], [43], [44]. Bosutinib makes only very limited contact with the activation loop in our structure, and aligning the structure of Abl bound to dasatinib onto our structure suggests that both conformations of the DFG motif are equally well accommodated by bosutinib (Figure 3A). The aspartate residue of the DFG motif is protonated in the DFG-Out conformation, and low pH has been shown to stabilize the DFG-Out conformation of Abl [45]. The fact that our crystals of the Abl:bosutinib complex were obtained at pH 5.5, combined with the absence of phosphorylation on the activation loop - a posttranslational modification that stabilizes the activation loops of many kinases in the active conformation [46], [47] - likely explains the DFG-Out conformation observed in our structure.

Phosphorylation on Tyr 393 in the activation loop of Abl stabilizes the DFG-In conformation (Figure 3A), and severely interferes with the binding of imatinib, which binds exclusively to the DFG-Out conformation of Abl [40]. To test whether bosutinib can bind to the active conformation of Abl, in addition to the DFG-Out conformation observed in our structure, we measured the binding of bosutinib to Abl that was phosphorylated on the activation loop. Abl kinase domain was phosphorylated using catalytic amounts of the Src kinase Hck [40]. The binding constant of bosutinib for phosphorylated Abl was indistinguishable from unphosphorylated Abl (Figure 3B). This indicates that bosutinib, unlike imatinib, can bind to the DFG-In conformation of Abl as well as the DFG-Out conformation of Abl observed in the structure. Apparently, like other next-generation BCR-Abl inhibitors, bosutinib binds to the kinase domain of Abl with less stringent conformational requirements than imatinib, an observation that has been used to explain the higher affinity of these compounds.

### The nitrile group of bosutinib affords a sensitive vibrational probe of the local environment in the ATP-binding site

While the kinase domains of Abl and Src share ∼48% sequence identity, the residues projecting into the ATP-binding site are completely conserved between the two proteins. High sequence conservation of the ATP-binding site is characteristic of protein kinases and contributes to the difficulty of developing selective kinase inhibitors [48].

We wondered how similar Src and Abl actually are in terms of the physical environment of the ATP-binding site. The nitrile group of bosutinib happens to possess favorable properties for addressing this question, as its vibrational absorption occurs in a region of the infrared spectrum that is uncluttered by contributions from protein groups and is highly sensitive to the local electric field through the vibrational Stark effect [27].

**Figure 4:**
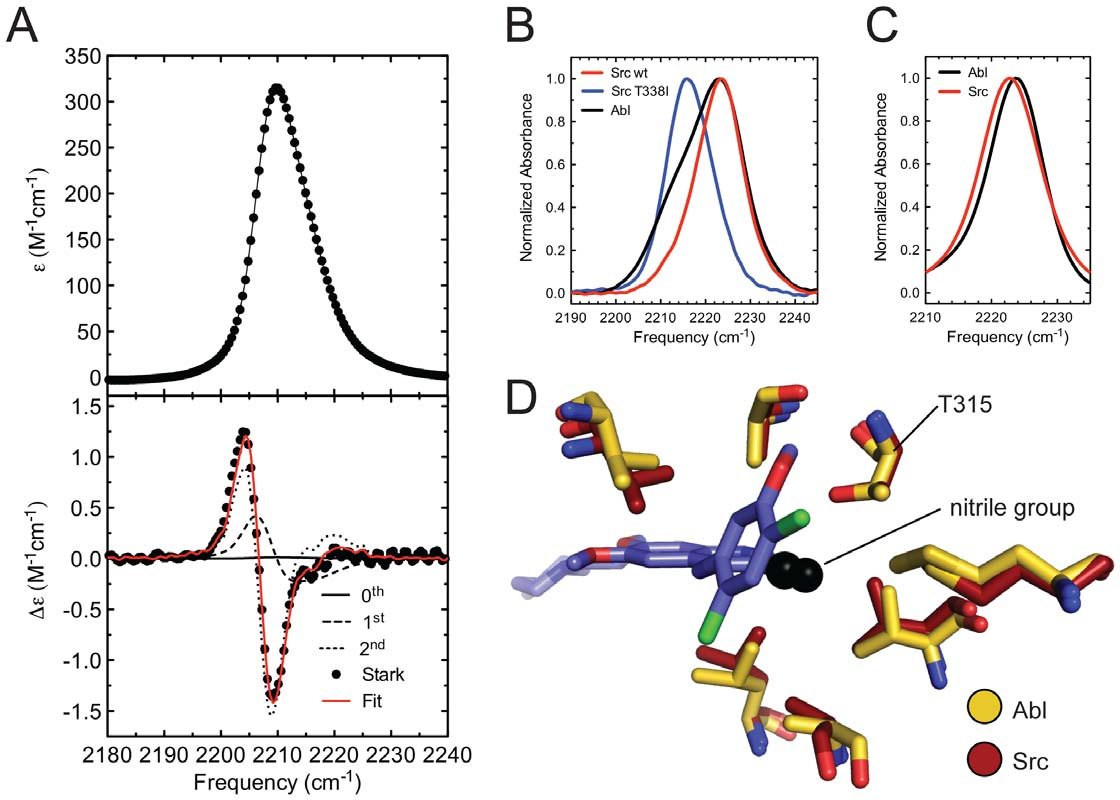
The nitrile group of bosutinib and the bosutinib isomer probe electrostatics in the ATP-binding site. A) Infrared absorbance (top) and Stark (bottom) spectra of 50 mM bosutinib in 1-propanol, measured at 77 K. A numerical fit to the Stark spectrum, from which the linear Stark tuning rate was derived, is shown in red. The numerical fit is a weighted sum of the derivatives of the absorption spectrum, and the individual fit components are shown as thin lines. B) The nitrile stretch region of infrared absorbance spectra of bosutinib bound to the kinase domains of Abl (black), Src (red) and the Src T338I mutant (blue). C) Infrared spectra of the bosutinib isomer bound to Abl (black) and Src (red). D) The residues that comprise the ATP-binding site near the nitrile of bosutinib (black) are shown for our structure of Abl bound to bosutinib (yellow) and for that of Src bound to dasatinib (pdb code 3G5D, dark red).

The vibrational Stark effect allows shifts in the absorbance of a vibrational probe, 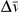, to be related to changes in the projection of the local electric field along the probe axis, 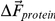, through the relationship 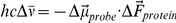, where *h* is Planck's constant, *c* is the speed of light and 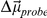 is the linear Stark tuning rate of the vibrational probe. To calibrate the sensitivity of the bosutinib nitrile to electric fields we performed vibrational Stark spectroscopy measurements, where the linear Stark tuning rate is determined by applying an external electric field across the sample and measuring the effect on the vibrational absorption (Figure 4A) [27]. The linear Stark tuning rate of bosutinib is 0.87 cm^−1^/(MV/cm), which is similar to the value for other aromatic nitriles [27], [49]. Mutations in proteins have been shown to result in changes in electric field as large as 10–20 MV/cm, producing peak shifts of nitrile probes of up to 15 cm^−1^, which can be routinely measured [50], [51].

We measured the vibrational absorption of bosutinib when bound to the kinase domains of Abl, Src, and the Src T338I mutant using fourier transform infrared (FTIR) spectroscopy (Figure 4B). The nitrile stretching band of bosutinib is very similar when bound to Abl and Src, although a shoulder in the Abl spectrum complicates the determination of the precise peak position. In contrast, the nitrile band is shifted ∼7 cm^−1^ to the red in the case of the Src T338I mutant. This shift corresponds to a difference in electric field of 8 MV/cm or 3 kT/eÅ, indicating that the nitrile group experiences a completely different environment in this mutant. A possible explanation for this observation is that the mutation of the gatekeeper removes a repulsive electrostatic interaction between the nitrile group and the sidechain hydroxyl group of the gatekeeper. A change in the binding mode of the drug is also a possible explanation, although it should be noted that while bosutinib binds the T338I mutant much more weakly than wildtype Src, it still binds with nanomolar affinity (see Figure 3B) and the binding mode is likely similar.

We also measured the nitrile vibrational frequency of the bosutinib isomer, which possesses a similar linear Stark tuning rate to bosutinib (Figure S3), bound to Abl and Src (Figure 4C). For this compound both IR spectra display single peaks in the nitrile stretch region, and the high quality of the spectra allows the peak positions to be determined to within ∼0.1 cm^−1^. The nitrile bands differ by 1.1 cm^−1^, corresponding to a difference in electric field experienced by the nitrile of 1.4 MV/cm or 0.6 kT/eÅ. This difference in the field can be directly converted into a measure of how favorable the electrostatic environment of the ATP-binding site is for the nitrile group of the bosutinib isomer. Nitrile groups possess a dipole moment of ∼2–4 Debye or 0.4–0.8 eÅ, and the difference in field of 0.6 kT/eÅ translates into a difference in electrostatic energy of 0.25–0.5 kT for the nitrile group of bosutinib in Src and Abl, indicating that the electrostatic environment is slightly more favorable for the nitrile in Src than in Abl.

While this difference is relatively small, it is nonetheless on a scale that is energetically significant, which is remarkable given that identical residues make up the ATP-binding sites of Src and Abl (Figure 4D). Assuming such differences are representative of other locations in the ATP-binding site, one can conclude that an inhibitor with optimal electrostatic properties could possess significant selectivity between Src and Abl, despite the conservation of their ATP-binding sites. It will be interesting to see the extent to which the environment of the ATP-binding site varies across more distantly related protein kinases, and we are now pursuing experiments to address this.

### Conclusion

Clinical resistance to kinase inhibitors is currently the primary problem facing the treatment of CML. Our structure explains the activity of bosutinib against imatinib resistant mutants of Abl, and should help to rationalize patterns of resistance that may yet emerge from the use of bosutinib in the clinic. While bosutinib, like the three currently approved inhibitors of BCR-Abl, is inactive against the common T315I mutation, our results suggest that the related 4-anilinoquinazolines are not affected by this mutation, and might yield an effective remedy for this form of BCR-Abl.

The high degree of sequence conservation in the ATP-binding sites of protein kinases hampers the development of selective kinase inhibitors. We have shown that nitrile-bearing inhibitors like bosutinib and the bosutinib isomer can be used to study electrostatic differences in the ATP-binding sites of kinases. The closely related kinases Src and Abl have identical ATP-binding site sequences, but nonetheless display distinct electrostatics. More distantly related kinases are likely to have much larger differences in electrostatics, and a thorough understanding of such differences might allow for the rational design of selective inhibitors whose electrostatic properties are tailored to the electrostatics of the ATP-binding site they are intended to bind.

## Supporting Information

Figure S1 **Activity of bacterially expressed Abl kinase domain.** Bacterially expressed Abl is catalytically active and inhibited by imatinib. Kinase activity was measured using a coupled kinase assay in which the production of ADP by the kinase is linked to the oxidation of NADH by pyruvate kinase and lactate dehydrogenase^1^.
(DOC)

Figure S2 **NMR experiments on bosutinib and the bosutinib isomer.** A) The structure of bosutinib and a putative structure for the bosutinib isomer are shown. The blue numbers on the bosutinib structure represent the five aromatic proton-carbon pairs. The numbers on the aniline ring of the bosutinib isomer are ^13^C chemical shifts. B) NMR spectra. In the top left panel, ^1^H-^13^C HSQC spectra of bosutinib and the bosutinib isomer are shown. The thick black lines connect the peaks that arise from the equivalent proton-carbon pairs in the two compounds. The thin gray lines are intended to guide the eye to the corresponding peaks in the 1-dimensional spectra. The peaks for the five aromatic proton-carbon pairs in authentic bosutinib are indicated with large blue numbers. These putative assignments are based on ^13^C chemical shift predictions. The bottom panel shows the ^1^H NMR spectra of both compounds. The peak located at 7.34 ppm in the bosutinib isomer sample, which integrates to 2, is indicated. The colored numbers directly next to the peaks are the peak integrations. The panel on the upper right shows the aromatic region of the ^13^C NMR spectrum of the bosutinib isomer. The peak located at 123 ppm, which displays an integrated intensity of 2, is indicated.
(DOC)

Figure S3 **Vibrational absorption (top) and Stark (bottom) spectra of 50 mM bosutinib isomer in 1-propanol at 77 K.** A numerical fit to the Stark spectrum, from which the linear Stark tuning rate was derived, is shown in red. The numerical fit is a weighted sum of the derivatives of the absorption spectrum, and the individual fit components are shown as thin lines. The value of the linear Stark tuning rate is 0.74 cm^−1^/(MV/cm).
(DOC)
