# Supplementary material for: Structural and Spectroscopic Analysis of the Kinase Inhibitor Bosutinib and an Isomer of Bosutinib Binding to the Abl Tyrosine Kinase Domain
Source: PLoS One. 2012 Apr 6;7(4):e29828. doi: 10.1371/journal.pone.0029828 (PMC3320885; doi:10.1371/journal.pone.0029828)
Supplement: Figure S1 — Activity of bacterially expressed Abl kinase domain. Bacterially expressed Abl is catalytically active and inhibited by imatinib. Kinase activity was measured using a coupled kinase assay in which the production of ADP by the kinase is linked to the oxidation of NADH by pyruvate kinase and lactate dehydrogenase1. (DOC) [file pone.0029828.s001.doc]

**Structural and spectroscopic analysis of the kinase inhibitor bosutinib and an isomer of bosutinib binding to the Abl tyrosine kinase domain**

Nicholas M. Levinson* and Steven G. Boxer

Department of Chemistry, Stanford University, Stanford CA 94305-5080

*Email: nickl@stanford.edu

**Supporting Information**


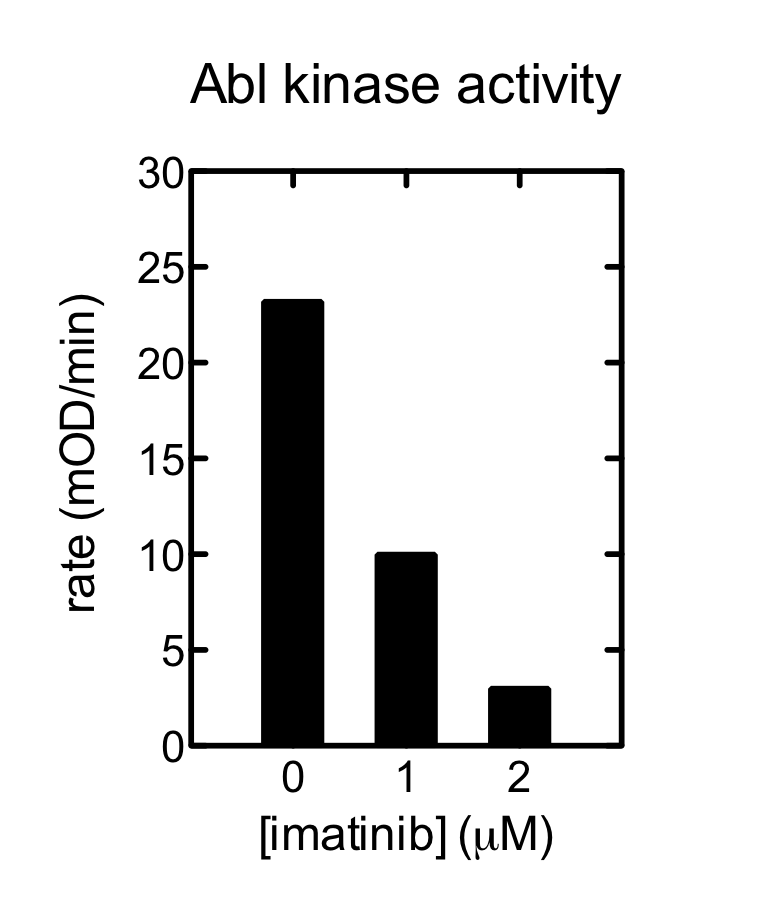


**Figure S1.** Activity of bacterially expressed Abl kinase domain. Bacterially expressed Abl is catalytically active and inhibited by imatinib. Kinase activity was measured using a coupled kinase assay in which the production of ADP by the kinase is linked to the oxidation of NADH by pyruvate kinase and lactate dehydrogenase^1^.

**References**

(1) Seeliger, M. A.; Young, M.; Henderson, M. N.; Pellicena, P.; King, D. S.; Falick, A. M.; Kuriyan, J. *Protein Sci* **2005**, *14*, 3135.
