# Supplementary material for: Structural and Spectroscopic Analysis of the Kinase Inhibitor Bosutinib and an Isomer of Bosutinib Binding to the Abl Tyrosine Kinase Domain
Source: PLoS One. 2012 Apr 6;7(4):e29828. doi: 10.1371/journal.pone.0029828 (PMC3320885; doi:10.1371/journal.pone.0029828)
Supplement: Figure S2 — NMR experiments on bosutinib and the bosutinib isomer. A) The structure of bosutinib and a putative structure for the bosutinib isomer are shown. The blue numbers on the bosutinib structure represent the five aromatic proton-carbon pairs. The numbers on the aniline ring of the bosutinib isomer are 13C chemical shifts. B) NMR spectra. In the top left panel, 1H-13C HSQC spectra of bosutinib and the bosutinib isomer are shown. The thick black lines connect the peaks that arise from the equivalent proton-carbon pairs in the two compounds. The thin gray lines are intended to guide the eye to the corresponding peaks in the 1-dimensional spectra. The peaks for the five aromatic proton-carbon pairs in authentic bosutinib are indicated with large blue numbers. These putative assignments are based on 13C chemical shift predictions. The bottom panel shows the 1H NMR spectra of both compounds. The peak located at 7.34 ppm in the bosutinib isomer sample, which integrates to 2, is indicated. The colored numbers directly next to the peaks are the peak integrations. The panel on the upper right shows the aromatic region of the 13C NMR spectrum of the bosutinib isomer. The peak located at 123 ppm, which displays an integrated intensity of 2, is indicated. (DOC) [file pone.0029828.s002.doc]

**Structural and spectroscopic analysis of the kinase inhibitor bosutinib and an isomer of bosutinib binding to the Abl tyrosine kinase domain**

Nicholas M. Levinson* and Steven G. Boxer

Department of Chemistry, Stanford University, Stanford CA 94305-5080

*Email: nickl@stanford.edu

**Supporting Information**

**NMR spectroscopy on bosutinib and the bosutinib isomer**

As described in the main text, the ^1^H NMR spectra of the compounds we purchased from LC Labs and Tocris Bioscience are strikingly different in the aromatic region. Five unique aromatic proton peaks are expected from the chemical structure of bosutinib, three on the quinoline ring and two on the aniline ring (Figure S2A). The ^1^H NMR spectra of both compounds, measured at 20 mM concentration in DMSO-*d_6_* at room temperature, instead display four peaks in the aromatic region (Figure S2B, bottom panel). However, for both compounds, one of the four peaks displays an integrated intensity of two, indicating it arises from two protons. These peaks are found at 7.34 ppm for the LC Labs compound, and 7.29 ppm for the Tocris Bioscience compound.

By themselves, these spectra do not contain sufficient information to confirm which compound is correct. We therefore performed ^1^H-^13^C Heteronuclear Single Quantum Coherence (HSQC) experiments, which correlates each proton with the carbon nucleus to which it is covalently attached (Figure S2B, top left panel). In the case of the compound from Tocris Bioscience five ^13^C crosspeaks are observed (shown in blue in Figure S2B), with the proton peak at 7.29 ppm having two ^13^C crosspeaks at 110 and 114 ppm. This indicates that the larger peak in the ^1^H NMR spectrum arises from two chemically non-equivalent protons that happen to have similar chemical shifts.

In contrast, the HSQC spectrum of the compound from LC Labs displays only four crosspeaks. The ^13^C crosspeak for the proton nucleus at 7.34 ppm is found at 123 ppm. This indicates that two protons, sharing the same chemical shift, are covalently bonded to two carbon nuclei that also share the same chemical shift. To further demonstrate the existence of aromatic carbon nuclei with identical chemical shifts we measured the ^13^C carbon NMR spectrum of the LC Labs compound (Figure S2B, top right panel). Instead of the expected 16 unique chemical shifts for aromatic carbons expected from the structure of bosutinib, only 14 peaks are observed. Two of these peaks display an integrated intensity close to two, indicating they arise from pairs of carbon nuclei in identical environments. The chemical shift of one of these peaks is 123 ppm, identical to the single crosspeak observed in the HSQC spectrum.

Note that of the five crosspeaks observed in the HSQC spectrum of the Tocris Bioscience compound, three have ^13^C chemical shifts that closely match crosspeaks seen in the spectrum of the LC Labs compound (linked by thick black lines in the figure), while the remaining two crosspeaks have ^13^C chemical shifts that are totally different from the fourth crosspeak in the LC Labs spectrum. Since the quinoline ring possesses three protons and the aniline ring two, this observation supports the findings from the x-ray data that the difference between the two compounds is on the aniline ring.

**
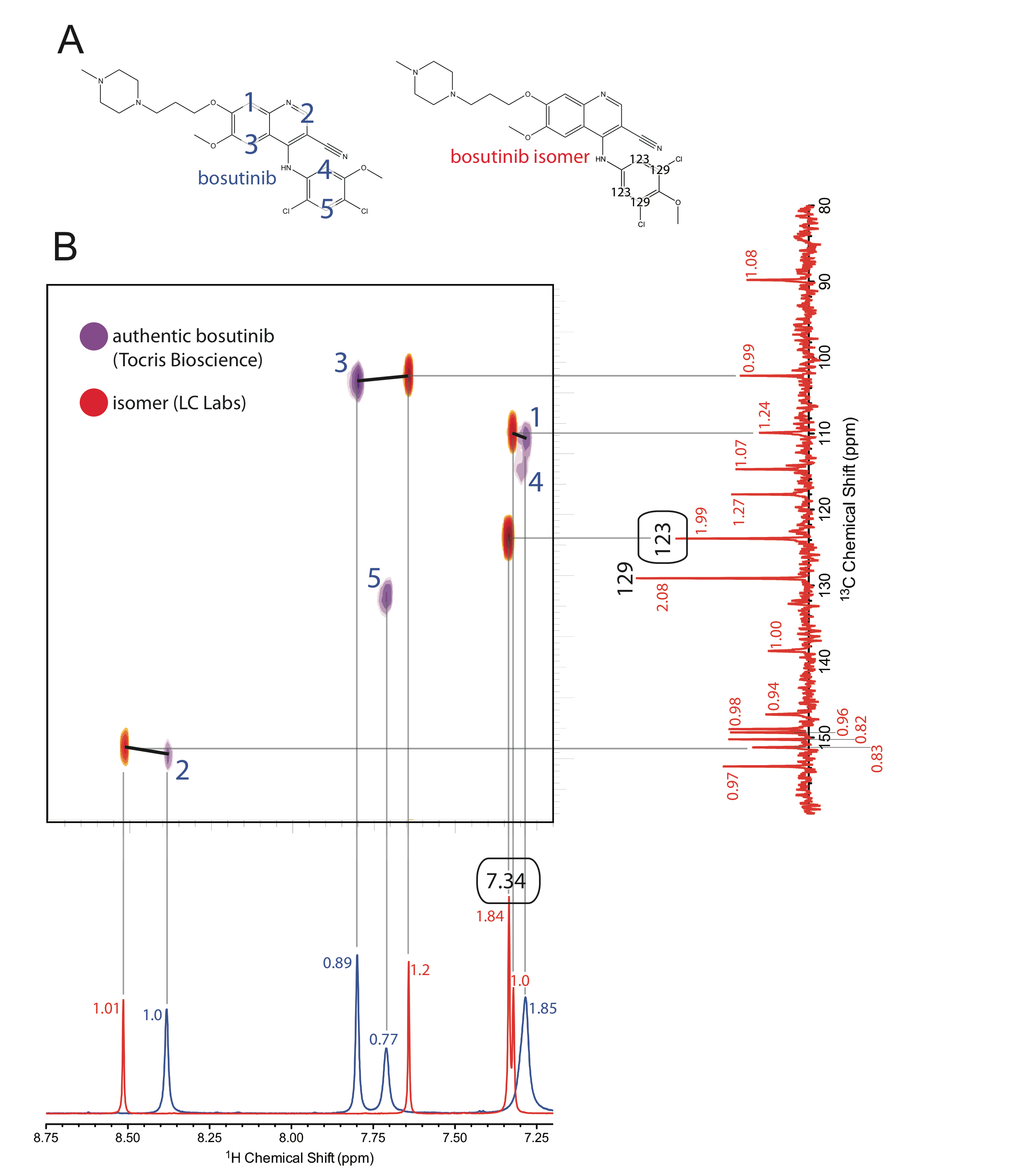
Figure S2.** NMR experiments on bosutinib and the bosutinib isomer. A) The structure of bosutinib and a putative structure for the bosutinib isomer are shown. The blue numbers on the bosutinib structure represent the five aromatic proton-carbon pairs. The numbers on the aniline ring of the bosutinib isomer are ^13^C chemical shifts. B) NMR spectra. In the top left panel, ^1^H-^13^C HSQC spectra of bosutinib and the bosutinib isomer are shown. The thick black lines connect the peaks that arise from the equivalent proton-carbon pairs in the two compounds. The thin gray lines are intended to guide the eye to the corresponding peaks in the 1-dimensional spectra. The peaks for the five aromatic proton-carbon pairs in authentic bosutinib are indicated with large blue numbers. These putative assignments are based on ^13^C chemical shift predictions. The bottom panel shows the ^1^H NMR spectra of both compounds. The peak located at 7.34 ppm in the bosutinib isomer sample, which integrates to 2, is indicated. The colored numbers directly next to the peaks are the peak integrations. The panel on the upper right shows the aromatic region of the ^13^C NMR spectrum of the bosutinib isomer. The peak located at 123 ppm, which displays an integrated intensity of 2, is indicated.

While the NMR data described above do not prove that either compound is correct, the data for the LC Labs compound demonstrating the presence of symmetry are clearly incompatible with the correct structure. The most likely structure for the bosutinib isomer has the two chlorine atoms located in the meta position and the methoxy group in the para position on the aniline ring (Figure S2A). This would produce an aniline ring with C2 symmetry, explaining the existence of two pairs of carbon nuclei with identical chemical shifts and one pair of protons with identical chemical shifts. In contrast, the NMR data for the Tocris Bioscience compound are compatible with the correct structure, and, as described in the main text, our x-ray data for the drug bound to Abl reveal that the substituents on the aniline ring are all in the correct positions.

We have made a putative assignment of the five aromatic proton-carbon pairs, using the chemical shift prediction implemented in the ChemBioDraw Ultra software package (CambridgSoft). The calculated and observed ^13^C chemical shifts are shown in Table S1.

**Table S1. Calculated and observed ^13^**C chemical shifts for the five aromatic proton-carbon pairs of authentic bosutinib

| proton-carbon pair identifier shown in Figure S2 | 1 | 2 | 3 | 4 | 5 |
| --- | --- | --- | --- | --- | --- |
| calculated ^13^C chemical shift | 107 | 149 | 100 | 102 | 132 |
| observed ^13^C chemical shift | 109 | 150 | 102 | 113 | 130 |
