# Supplementary material for: Structural and Spectroscopic Analysis of the Kinase Inhibitor Bosutinib and an Isomer of Bosutinib Binding to the Abl Tyrosine Kinase Domain
Source: PLoS One. 2012 Apr 6;7(4):e29828. doi: 10.1371/journal.pone.0029828 (PMC3320885; doi:10.1371/journal.pone.0029828)
Supplement: Figure S3 — Vibrational absorption (top) and Stark (bottom) spectra of 50 mM bosutinib isomer in 1-propanol at 77 K. A numerical fit to the Stark spectrum, from which the linear Stark tuning rate was derived, is shown in red. The numerical fit is a weighted sum of the derivatives of the absorption spectrum, and the individual fit components are shown as thin lines. The value of the linear Stark tuning rate is 0.74 cm−1/(MV/cm). (DOC) [file pone.0029828.s003.doc]

**Structural and spectroscopic analysis of the kinase inhibitor bosutinib and an isomer of bosutinib binding to the Abl tyrosine kinase domain**

Nicholas M. Levinson* and Steven G. Boxer

Department of Chemistry, Stanford University, Stanford CA 94305-5080

*Email: nickl@stanford.edu

**Supporting Information**

**Stark spectroscopy of the bosutinib isomer**


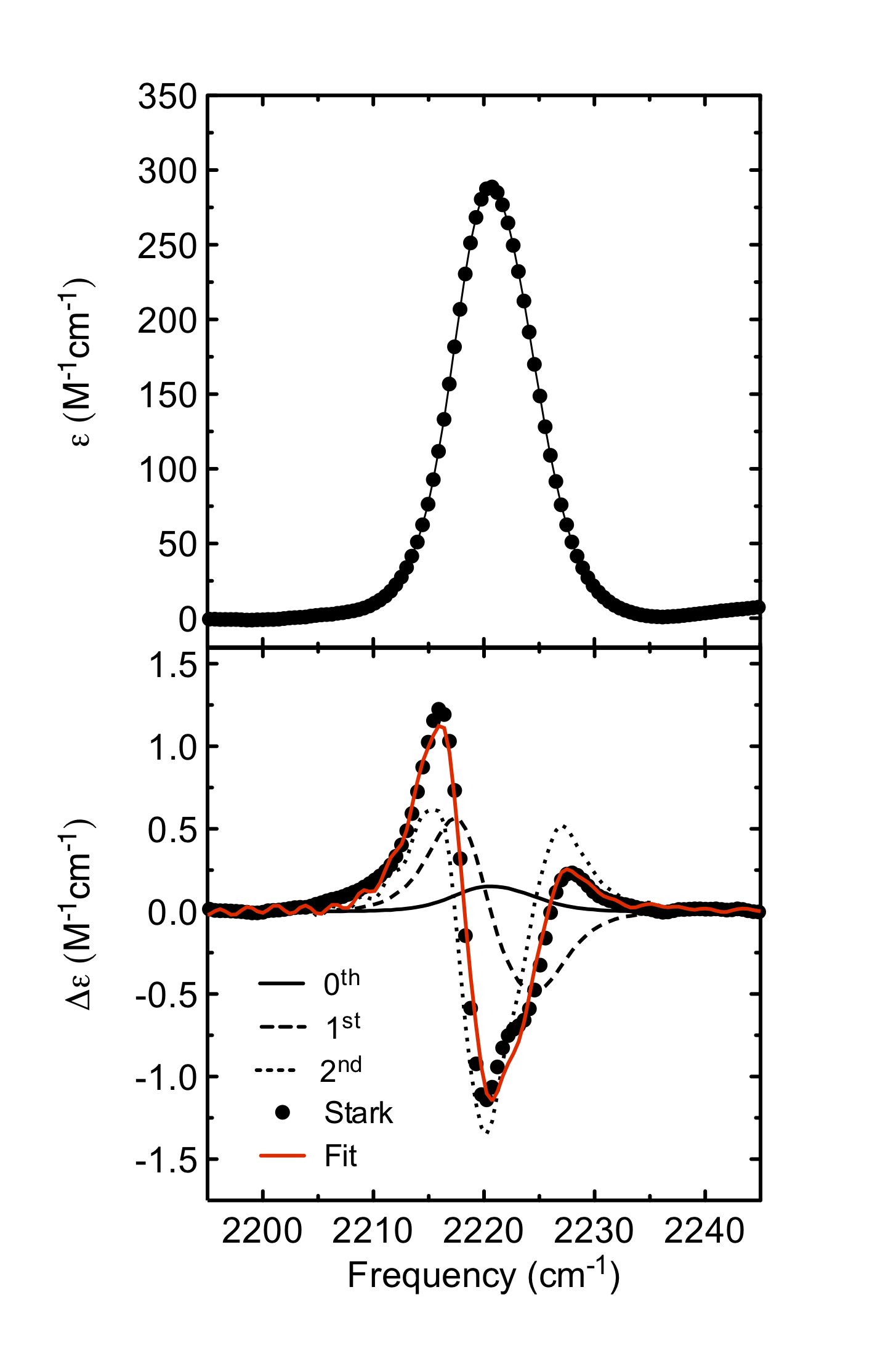


**Figure S3.** Vibrational absorption (top) and Stark (bottom) spectra of 50 mM bosutinib isomer in 1-propanol at 77K. A numerical fit to the Stark spectrum, from which the linear Stark tuning rate was derived, is shown in red. The numerical fit is a weighted sum of the derivatives of the absorption spectrum, and the individual fit components are shown as thin lines. The value of the linear Stark tuning rate is 0.74 cm^-1^/(MV/cm).
